# Physiological, biochemical and microstructural responses of the epiphytic orchid *Dendrobium chrysanthum* to drought stress

**DOI:** 10.3389/fpls.2026.1877050

**Published:** 2026-07-15

**Authors:** Zhe Yang, Lihui Peng, Lingzhi Wei, Feng Chen, Chenghao Zhu, Yujing Wei, Zhenhai Deng, Qiang Jiang, Xiao Wei, Danjuan Zeng, Tao Ding, Shengfeng Chai

**Affiliations:** 1 Key Laboratory of Plant Functional Phytochemicals and Sustainable Utilization, Guangxi Institute of Botany, Guilin, China; 2 College of Tourism and Landscape Architecture, Guilin University of Technology, Guilin, China; 3 Yachang Orchid National Nature Reserve Management Center, Baise, China

**Keywords:** antioxidant defense, *Dendrobium chrysanthum*, drought stress, epiphytic orchid, microstructure, photosynthetic physiology

## Abstract

To elucidate organ-level drought adaptation in epiphytic orchids, this study investigated the endangered species *Dendrobium chrysanthum* under well-watered control (CK), moderate drought (T1; 14 d of water withholding), and severe drought (T2; 28 d). Water status, photosynthetic physiology, microstructure, osmotic adjustment, antioxidant defense, and lipid peroxidation were analyzed. Relative water content (RWC) declined progressively in roots, pseudobulbs, and leaves, with pseudobulb RWC decreasing more markedly than leaf RWC under severe drought, suggesting that pseudobulbs may contribute to buffering leaf dehydration. Gas exchange was strongly suppressed: net photosynthetic rate (*P*_n_) decreased under T1 and became slightly negative under T2, while intercellular CO_2_ concentration (*C*_i_) first decreased and then increased, indicating a shift from stomatal limitation to non-stomatal inhibition. The slightly negative Pn under T2 suggests a transition toward a survival-maintenance state with limited carbon gain. Chlorophyll a, chlorophyll b, total chlorophyll, carotenoids, and the chlorophyll a/b ratio showed no significant differences among treatments, indicating maintenance of the pigment pool. Osmotic adjustment substances and antioxidant enzymes showed organ-specific responses, suggesting coordinated stem–leaf metabolic regulation and ROS-scavenging adjustment under drought. MDA content decreased under T1 but increased markedly under T2 in both leaves and pseudobulbs, indicating enhanced lipid peroxidation under severe drought; however, MDA levels remained lower in pseudobulbs than in leaves. *F*_v_/*F*_m_ was maintained under T1 but decreased under T2, and *Φ*_PSII_ declined progressively, reflecting partial photoinhibition. Anatomical changes, including increased root cortex thickness, reduced root xylem vessel diameter, and decreased pseudobulb tissue area fraction, further reflected structural adjustment associated with water conservation. Overall, *D. chrysanthum* appears to adopt a conservative drought-response strategy involving pseudobulb-associated water buffering, photosynthetic down-regulation, osmotic regulation, and organ-specific antioxidant adjustment.

## 1 Introduction

Water availability is a key ecological factor governing plant function, because it regulates cell turgor, stomatal movement, photosynthesis, and long-distance transport (Boyer, 1982; Fàbregas and Fernie, 2019). When water supply becomes limited, drought first restricts stomatal CO_2_ diffusion and then progressively impairs mesophyll carbon assimilation and PSII activity, leading to reduced photosynthetic performance and, under severe stress, photoinhibition (Flexas and Medrano, 2002; Baker, 2008). Drought is also closely associated with reactive oxygen species (ROS) accumulation, membrane lipid peroxidation, and disruption of cellular metabolic balance (Mittler, 2002; Gill and Tuteja, 2010). As climate change increases the frequency and duration of extreme drought events, understanding plant drought responses from water status, photosynthetic function, structural regulation, and antioxidant defense remains essential for evaluating stress adaptation and drought tolerance (Cook et al., 2020).

Epiphytes are specialized plants that grow on tree trunks or rock surfaces and depend largely on intermittent rainfall, fog, and atmospheric humidity rather than continuous soil water supply (Benzing, 1990; Zotz, 2016). This episodic water availability has promoted distinctive water-regulation strategies in epiphytic orchids, including velamen-bearing roots for rapid water absorption, enlarged pseudobulbs for water storage, and conservative regulation of photosynthesis (Stancato et al., 2001; Yang et al., 2016). Although pseudobulbs are generally recognized as water-storage organs, their potential contribution to drought-induced osmotic and antioxidant regulation remains less clearly resolved. Meanwhile, some epiphytic orchids show photosynthetic pathway plasticity along the C_3_-CAM continuum, which may further influence their responses to prolonged water deficit (Silvera et al., 2009; Winter, 2019). Therefore, an organ-level analysis that links water regulation, leaf photosynthetic function, and stem biochemical responses is needed to better understand drought adaptation in epiphytic orchids.

*Dendrobium chrysanthum* (Orchidaceae) is an epiphytic herb distributed mainly in tropical and subtropical montane forests of southwestern China and adjacent regions of the Indochina Peninsula, where it grows on tree trunks or rock surfaces subject to highly variable water availability. Its ornamental and medicinal value has increased commercial demand, while habitat loss and overcollection have contributed to the decline of wild populations (Swarts and Dixon, 2009; Teixeira da Silva and Ng, 2017). Existing studies on *D. chrysanthum* have focused largely on phytochemistry, tissue culture, and propagation, whereas drought-related responses have rarely been examined from an integrated organ-level perspective. More broadly, studies of other Dendrobium species suggest that pseudobulbs may contribute to water maintenance under drought (Yang et al., 2016), but whether pseudobulb tissues also participate in osmotic adjustment and antioxidant regulation during graded drought stress remains insufficiently quantified.

Here, *D. chrysanthum* was subjected to well-watered control, moderate drought, and severe drought treatments. We measured organ-level water status, leaf gas exchange, chlorophyll fluorescence and pigment contents, anatomical and stomatal traits, and osmotic adjustment, antioxidant enzyme activities, and MDA content in stems and leaves. We tested whether pseudobulbs primarily act as water-buffering organs and whether their stem tissues also show biochemical responses associated with osmotic and oxidative regulation under drought. We further examined whether photosynthetic inhibition shifts from stomatal to non-stomatal limitation as drought severity increases. These results are expected to clarify the drought-adaptation strategy of *D. chrysanthum* and provide a physiological basis for its cultivation and *ex situ* conservation.

## 2 Materials and methods

### 2.1 Study site and plant materials

The experiment was conducted in 2024 at the Guangxi Institute of Botany, Yanshan District, Guilin City, Guangxi Zhuang Autonomous Region, China (25°01′N, 110°17′E). The experimental site is located at an altitude of approximately 180 m and is characterized by a typical subtropical monsoon climate. The annual mean air temperature is 18.8 °C, with January being the coldest month (mean temperature 8.3 °C) and July the hottest month (mean temperature 28.3 °C). The annual precipitation ranges from 1900 to 2000 mm, more than 70% of which occurs from April to August. The mean annual relative humidity is approximately 78%, and the annual sunshine duration is about 1500 h. The experiment was carried out in autumn, from late September to late October, following the end of the local rainy season.

The plant materials used in this study were two-year-old healthy seedlings of *D. chrysanthum*, provided by the Orchid Propagation Center of the Guangxi Yachang Orchid National Nature Reserve, Baise, Guangxi, China. These seedlings were propagated through aseptic seed germination using fruits collected from wild *D. chrysanthum* plants. Plants with uniform growth and free from visible pests and diseases were selected for the experiment. Prior to the treatments, all plants were acclimated under natural conditions in a plastic greenhouse, where light intensity was maintained at approximately 20% of full sunlight, air temperature followed natural diurnal fluctuations, and relative humidity was kept at 60%–70%. During the acclimation period, plants were watered normally and fertilized regularly to ensure stable and homogeneous physiological status. Drought stress was imposed by withholding irrigation, and different stress levels were established according to the duration of water deprivation: CK (well-watered control, irrigated once every 3 d), T1 (water withheld for 14 d, moderate drought), and T2 (water withheld for 28 d, severe drought). The classification of drought intensity was determined by integrating visible wilting symptoms with changes in relative water content (RWC). All plants were grown in plastic pots 13 cm in diameter and 15 cm in depth, filled with a substrate mixture of bark, coconut coir, and perlite at a volume ratio of 3:1:1. Because this porous epiphytic substrate drains rapidly and may show heterogeneous water distribution, substrate moisture was not used as the primary criterion for drought classification. Instead, drought intensity was defined using plant water status, with SRWC as the primary indicator and LRWC together with visible wilting symptoms as supplementary indicators.

Drought treatments were imposed by withholding irrigation: for the severe drought group (T2), water was withheld beginning on September 1, 2024; for the moderate drought group (T1), plants were fully irrigated until September 15, 2024, after which water was withheld. Control plants (CK) were watered normally every 3 days to maintain substrate moisture close to saturation throughout the experiment. Drought intensity was graded using stem relative water content (SRWC) as the primary criterion—justified by the central role of pseudobulbs as water-storage organs in *Dendrobium* (Stancato et al., 2001; Yang et al., 2016)—supplemented by leaf wilting symptoms and leaf relative water content (LRWC). Treatment thresholds were defined prospectively before the experiment began: moderate drought (T1) when SRWC declined to 70%–85% of the well-watered control (CK); severe drought (T2) when SRWC fell below 65% of CK; borderline cases (SRWC 65%–70%) were resolved by integrating leaf wilting symptoms and LRWC (Lawlor and Cornic, 2002). The experiment lasted 28 d; physiological measurements were performed on September 28, 2024, after which root, stem, and leaf samples were immediately collected for biochemical analyses. At sampling, SRWC was 84.8% of CK under T1 and 64.9% of CK under T2, consistent with the predefined classification thresholds.

### 2.2 Measurement of relative water content

The water status of roots, stems, and leaves of *D. chrysanthum* was evaluated using relative water content (RWC). Four sets of fresh samples were collected, and their fresh weight (FW) was measured immediately. The samples were then immersed in distilled water at 4 °C for 24 h to obtain the turgid weight (TW), followed by oven-drying at 80 °C to constant weight to determine the dry weight (DW). Relative water content was calculated using the following equation:


$$RWC=\frac{FW-DW}{TW-DW}\times 100\%$$

### 2.3 Measurement of gas exchange parameters

Photosynthetic gas-exchange parameters of *D. chrysanthum* leaves were measured using a LI-6400 portable photosynthesis system (LI-COR, USA), including net photosynthetic rate (*P*_n_), stomatal conductance (*G*_s_), transpiration rate (*T*_r_), and intercellular CO_2_ concentration (*C*_i_). Fully expanded functional leaves of uniform growth were selected for measurement. Before data collection, leaves were pre-adapted for 20 min under the built-in red-blue light source at a PPFD of 500 μmol·m^-2^·s^-1^ to stabilize photosynthetic activity. Gas-exchange measurements were then conducted under open-system conditions with a flow rate of 0.5 L·min^-1^, a leaf chamber temperature of 28 °C, and a reference CO_2_ concentration of 400 μmol·mol^-1^. The selected PPFD was consistent with the reported light-saturation range for shade-adapted epiphytic orchids, typically around 400–600 μmol·m^-2^·s^-1^, and was further verified by preliminary light-response curve measurements conducted on control plants before the experiment (Rodrigues et al., 2013). Therefore, 500 μmol·m^-2^·s^-1^ was used as a near-saturating irradiance to ensure comparable gas-exchange measurements among treatments. All measurements were conducted between 09:00 and 11:00 to avoid midday photosynthetic depression. Four individual leaves (one per plant, four plants per treatment) were measured and values were averaged.

### 2.4 Measurement of chlorophyll fluorescence parameters

Chlorophyll fluorescence parameters were measured using a Mini-PAM portable fluorometer (WALZ, Germany). Prior to measurement, leaves were dark-adapted for 30 min to ensure full opening of PSII reaction centers. Parameters measured included minimal fluorescence (*F*_0_), maximal fluorescence (*F*_m_), maximum photochemical efficiency of PSII (*F*_v_/*F*_m_), and actual photochemical quantum yield of PSII (*Φ*_PSII_), which were used to assess PSII functional status under drought stress. Four replicates were measured per treatment, and mean values were used for analysis.

### 2.5 Measurement of photosynthetic pigments

Leaf chlorophyll content was determined spectrophotometrically. Four sets of fresh leaves were collected from each treatment, cut into small pieces, and extracted with 95% ethanol in the dark until the tissues were completely decolorized. The absorbance of the extracts was measured at 649 and 665 nm using a PerkinElmer Lambda 35 UV–Vis spectrophotometer (USA). The concentrations of chlorophyll a and chlorophyll b were calculated using the following equations:


$$Chl\;a=13.95\times A_{665}-6.88\times A_{649}$$

$$Chl\;b=24.96\times A_{649}-7.32\times A_{665}$$

Total chlorophyll content (mg·g^-1^) was calculated as:


$$Chl=\frac{C\times V\times N}{1000\times W}$$

where *C* is pigment concentration (mg·L^-1^), *V* is extraction volume (mL), *N* is the dilution factor, and *W* is leaf fresh weight (g).

### 2.6 Microstructural observations

Anatomical observations of roots, stems, and leaves, as well as leaf epidermal characteristics, were conducted using samples collected from plants previously subjected to photosynthetic measurements. For anatomical observations, four uniformly growing plants were selected from each treatment as independent biological replicates. From each plant, one root segment, one pseudobulb stem segment, and one fully expanded functional leaf were collected. Root and stem samples were taken from the middle portions of healthy roots and pseudobulbs, respectively, whereas leaf samples were taken from the middle region of fully expanded mature leaves located at the mid-portion of the plant. Samples were cut into small segments along the longitudinal or midrib direction and immediately fixed in FAA solution (formaldehyde–acetic acid–ethanol) for 24 h. Fixed samples were dehydrated through a graded ethanol series (2 h per step), cleared with xylene, and embedded in paraffin. Continuous sections with a thickness of 8 μm were prepared using a rotary microtome, stained with toluidine blue, mounted with neutral resin, and observed under a light microscope. Structural parameters of roots, stems, and leaves were quantified using CaseViewer image analysis software. Anatomical parameters measured included root cortex thickness (RCT), root xylem vessel diameter (RXVD), stem tissue area fraction (STAF), and leaf thickness (LT). STAF was defined as the percentage of the cross-sectional area occupied by cortical parenchyma cells relative to the total stem cross-sectional area, and was quantified using Image-Pro Plus software. For each biological replicate sample, measurements were taken from ten randomly selected fields of view. These field-level measurements were treated as technical subsamples and averaged to obtain one value per biological replicate before statistical analysis.

Leaf epidermal structural characteristics were examined using four plants per treatment as independent biological replicates. One healthy, fully expanded mature leaf was sampled from each plant. Small segments (approximately 4 mm × 4 mm) were excised along the direction of the main vein for subsequent microscopic observation. Samples were fixed in 2.5% glutaraldehyde at 4 °C for 24 h, dehydrated through an ethanol gradient (30%, 50%, 70%, 90%, and 100%; 30 min per step), and dried using a critical point dryer. Dried samples were sputter-coated with gold for approximately 90 s and observed using a scanning electron microscope (ZEISS EVO18). For each biological replicate sample, five randomly selected fields of view were photographed and measured. The values from these five fields were averaged to obtain one biological replicate value before statistical analysis. Stomatal density (SD), stomatal length (SL), and stomatal width (SW) were analyzed using Image-Pro Plus 6.0 software. Stomatal area (SA) was calculated using the ellipse area formula:


$$SA=\frac{\pi ab}{4}$$

where *a* and *b* represent stomatal length and width, respectively.

To decouple purely physiological regulation (aperture control) from developmental patterning (density changes), all microstructural and epidermal measurements were strictly restricted to mature leaves that had fully expanded prior to the initiation of the drought treatments; no newly emerged leaves formed during the 28-day experimental period were used for anatomical analysis.

### 2.7 Measurement of osmotic adjustment substances

Soluble sugar (SS), soluble protein (SP), and starch (ST) contents were determined in stems and leaves using spectrophotometric colorimetric methods, with four plants per treatment serving as independent biological replicates. SS content was measured by the anthrone–sulfuric acid method (absorbance at 620 nm). SP content was determined by the Coomassie Brilliant Blue G-250 (Bradford) method (absorbance at 595 nm); this method was preferred over the BCA assay because it is less susceptible to interference from the reducing sugars and phenolic compounds commonly present in orchid tissues (Rodrigues et al., 2013). For ST, soluble sugars were first removed by repeated washing with 80% ethanol; the residue was hydrolyzed with perchloric acid and glucose content was measured by the anthrone method, from which ST content was calculated.

### 2.8 Measurement of antioxidant enzyme activities

Antioxidant enzyme activities were determined in stems and leaves using spectrophotometric assays. Four biological replicates were used for each treatment. Fresh samples were homogenized in pre-chilled extraction buffer under ice-bath conditions and centrifuged, and the supernatant was used for enzyme activity assays. Superoxide dismutase (SOD) activity was determined using the xanthine–xanthine oxidase system, which generates superoxide radicals (O_2_^-^·). These radicals react with WST-8 to produce a water-soluble formazan dye with absorbance at 450 nm. SOD scavenges O_2_^-^·, thereby inhibiting formazan formation, and the decrease in color intensity is inversely proportional to SOD activity. Catalase (CAT) activity was measured based on its ability to decompose hydrogen peroxide (H_2_O_2_), which exhibits characteristic absorbance at 240 nm. The decline in absorbance over time was used to calculate CAT activity. Peroxidase (POD) activity was determined by measuring the rate of oxidation of a specific substrate catalyzed by POD in the presence of H_2_O_2_, with absorbance recorded at 470 nm. Final enzyme activities are expressed as U·g^-1^ FW (SOD, POD) or nmol·min^-1^·g^-1^ FW (CAT), where one unit (U) is defined as the amount of enzyme causing 50% inhibition of the formazan reaction (SOD) or producing a 0.01 change in absorbance per minute (POD).

### 2.9 Measurement of lipid peroxidation

Lipid peroxidation was evaluated by measuring malondialdehyde (MDA) content using the thiobarbituric acid (TBA) method, with four biological replicates per treatment. Reactive oxygen species attack unsaturated fatty acids in membrane lipids, leading to the formation of lipid peroxides that subsequently decompose into various products, among which MDA is a representative marker. MDA reacts with TBA to form a red-colored complex with maximum absorbance at 532 nm. Absorbance at 600 nm was also measured to correct for nonspecific turbidity, and MDA content was calculated based on the difference between absorbance values at 532 nm and 600 nm.

### 2.10 Data analyses

Raw data were organized and preprocessed in Microsoft Excel 2019. Prior to analysis, data for each variable were tested for normality using the Shapiro–Wilk test and for homogeneity of variance using Levene’s test. No obvious deviations from normality or homogeneity of variance were detected based on the Shapiro–Wilk and Levene’s tests. One-way ANOVA was therefore used to evaluate treatment effects, followed by Duncan’s multiple range test for *post-hoc* pairwise comparisons when ANOVA revealed a significant effect (*P* < 0.05). Pearson’s bivariate correlation analysis was conducted to examine relationships among physiological, biochemical, and microstructural parameters. All statistical analyses were performed in SPSS Statistics 27. Figures were prepared using Origin 2021. Data are presented as mean ± standard deviation (*n* = 4).

## 3 Results

### 3.1 Relative water content

**Figure 1:**
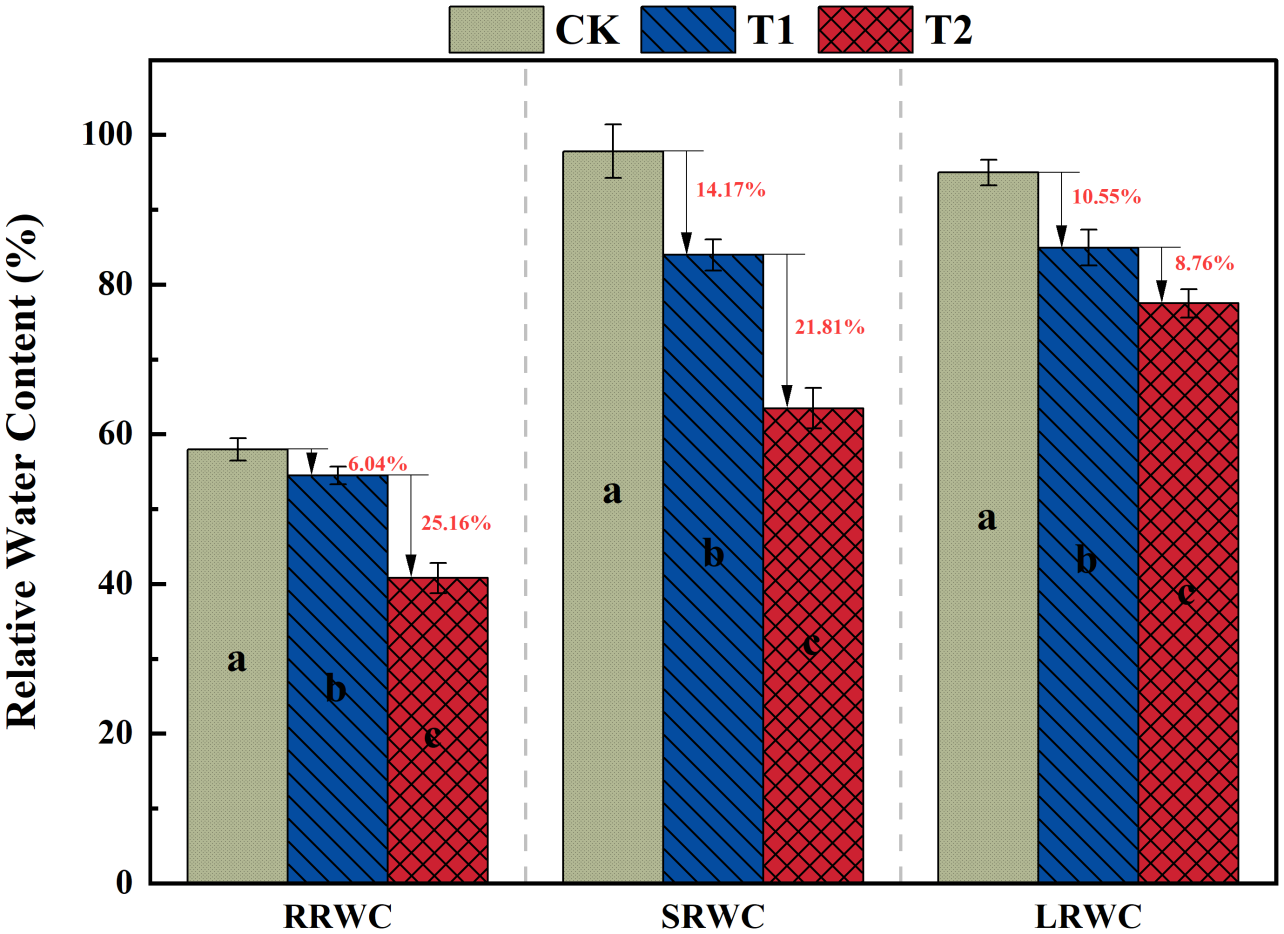
Changes in relative water content of roots, stems and leaves of *D. chrysanthum* under different drought stress treatments. Data are presented as mean ± standard deviation (*n* = 4). Different colored bars represent the control (CK), moderate drought stress (T1) and severe drought stress (T2), respectively. Different lowercase letters indicate significant differences among treatments at *P* < 0.05 within the same parameter. RRWC, root relative water content; SRWC, stem relative water content; LRWC, leaf relative water content.

**Figure 2:**
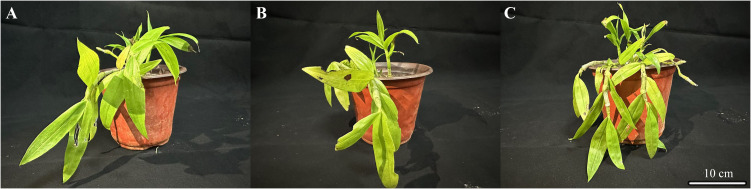
Phenotypic responses of *D. chrysanthum* plants under different drought stress treatments. A, B and C represent the control (CK), moderate drought stress (T1) and severe drought stress (T2), respectively. Scale bar = 10 cm.

RWC in roots, pseudobulbs, and leaves of *D. chrysanthum* decreased progressively with increasing drought intensity (**Figure 1**). Compared with CK, the RWC of all three organs declined significantly under T1 and decreased further under T2. From CK to T2, RWC decreased by approximately 29.7% in roots, 35.1% in pseudobulbs, and 18.4% in leaves, with the largest overall reduction observed in pseudobulbs and the smallest reduction in leaves. Between T1 and T2, stem RWC continued to decline markedly, whereas leaf RWC showed a smaller further decrease. Although some variation was observed among individual plants, the overall drought-induced decline in RWC was consistent across roots, pseudobulbs, and leaves. Phenotypically, CK plants grew vigorously with erect leaves and plump pseudobulbs, whereas T2 plants exhibited drooping and wilting leaves together with visible shrinkage and wrinkling of pseudobulbs (**Figure 2**).

### 3.2 Gas exchange parameters

Under different drought stress conditions, all leaf gas-exchange parameters of *D. chrysanthum* exhibited significant changes (**Table 1**). Compared with the CK, *P*_n_, *G*_s_, *C*_i_, and *T*_r_ were all significantly reduced under the T1 treatment. With further intensification of drought, *P*_n_, *G*_s_, and *T*_r_ declined further under T2 and were significantly lower than those under both CK and T1. In contrast, *C*_i_ showed a distinct response pattern: under T2, *C*_i_ increased significantly, being markedly higher than that under T1 and recovering to levels close to or even exceeding those of CK. Overall, drought stress markedly inhibited leaf gas exchange in *D. chrysanthum*, whereas *C*_i_ displayed a response different from the other gas-exchange parameters under severe drought conditions.

**Table 1:** Changes in gas exchange parameters of leaves in *D. chrysanthum* under different drought stress treatments.

Treatment	*P*_n_ (μmol·m^-2^·s^-1^)	*G*_s_ (mol·m^-2^·s^-1^)	*C*_i_ (μmol·mol^-1^)	*T*_r_ (mmol·m^-2^·s^-1^)
CK	2.741 ± 0.157^a^	0.023 ± 0.002^a^	417.75 ± 19.07^b^	0.261 ± 0.017^a^
T1	1.081 ± 0.098^b^	0.012 ± 0.001^b^	323.01 ± 12.53^c^	0.121 ± 0.007^b^
T2	-0.031 ± 0.026^c^	0.003 ± 0.001^c^	499.40 ± 26.12^a^	0.021 ± 0.002^c^

Different lowercase letters within the same column indicate significant differences among treatments at *P* < 0.05, whereas the same lowercase letters indicate no significant difference. Data are presented as mean ± standard deviation. *P*_n_, net photosynthetic rate; *G*_s_, stomatal conductance; *C*_i_, intercellular CO_2_ concentration; *T*_r_, transpiration rate.

### 3.3 Chlorophyll fluorescence

Under different drought stress conditions, the chlorophyll fluorescence parameters of *D. chrysanthum* leaves exhibited distinct changes (**Table 2**). Compared with the CK, *F*_0_ increased under the T1 treatment, whereas *F*_m_ showed a decreasing trend; under T2, *F*_0_ increased further and *F*_m_ declined markedly. Meanwhile, *F*_v_/*F*_m_ showed no obvious difference from CK under T1 but decreased significantly under T2, while *Φ*PSII displayed a progressive decline with increasing drought severity.

**Table 2:** Changes in chlorophyll fluorescence parameters of leaves in *D. chrysanthum* under different drought stress treatments.

Treatment	*F*_0_	*F*_m_	*F*_v_/*F*_m_	*Φ*_PSII_
CK	216.25 ± 9.38^b^	874.25 ± 12.05^a^	0.75 ± 0.02^a^	0.32 ± 0.02^a^
T1	233.75 ± 8.11^ab^	793.00 ± 26.20^b^	0.71 ± 0.02^a^	0.29 ± 0.02^a^
T2	246.80 ± 15.60^a^	725.40 ± 38.00^c^	0.66 ± 0.03^b^	0.23 ± 0.02^b^

Different lowercase letters within the same column indicate significant differences among treatments at *P* < 0.05, whereas the same lowercase letters indicate no significant difference. Data are presented as mean ± standard deviation. *F*_0_, fluorescence origin; *F*_m_, fluorescence maximum; *F*_v_/*F*_m_, maximal quantum yield of PSII photochemistry; *Φ*PSII, effective quantum yield of PSII photochemistry.

### 3.4 Photosynthetic pigment

**Figure 3:**
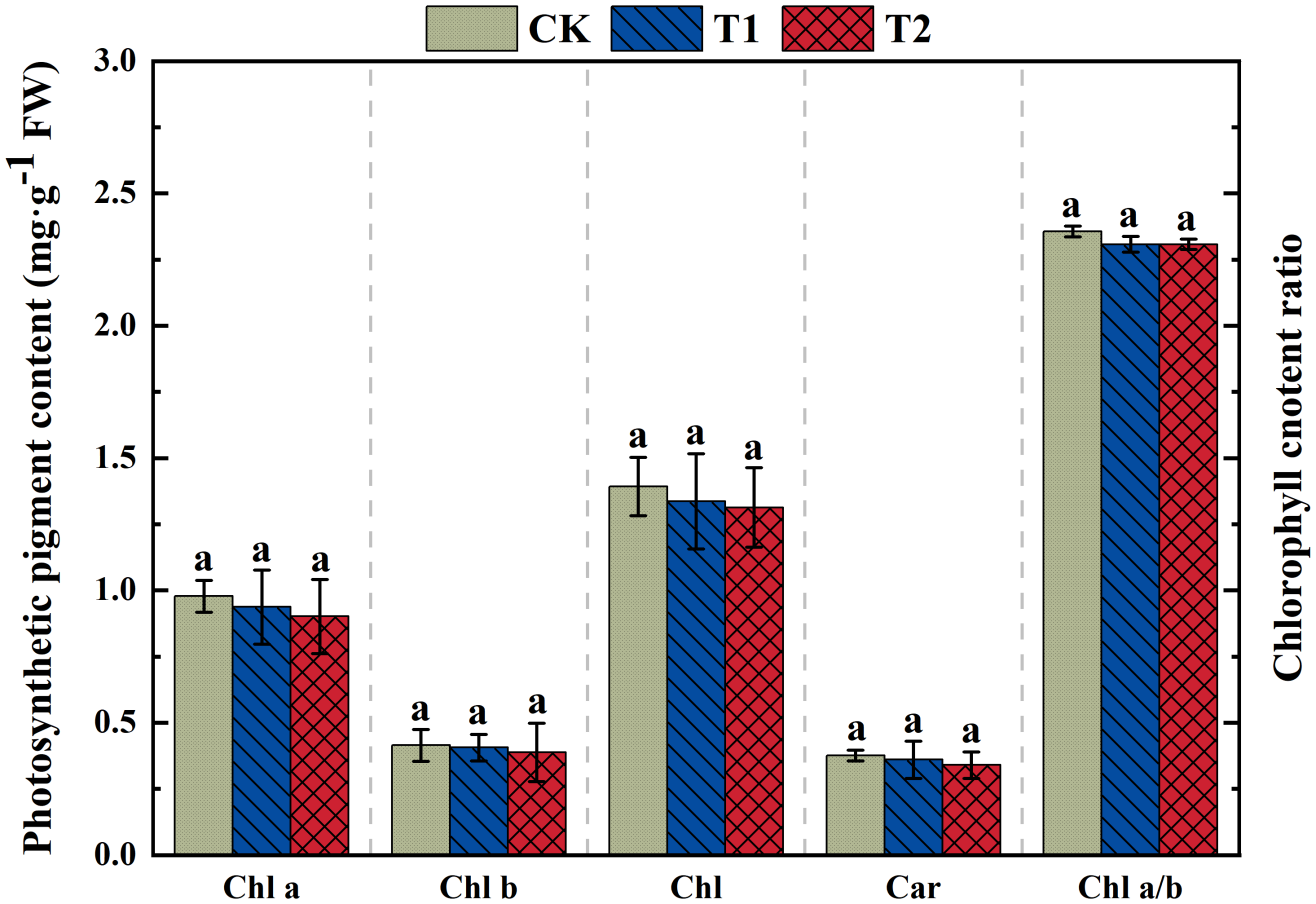
Changes in photosynthetic pigment contents and chlorophyll a/b ratio in leaves of *D. chrysanthum* under different drought stress treatments. Data are presented as mean ± standard deviation (*n* = 4). Different colored bars represent the control (CK), moderate drought stress (T1) and severe drought stress (T2), respectively. Different lowercase letters indicate significant differences among treatments at *P* < 0.05 within the same parameter; the same letters indicate no significant difference. Chl a, chlorophyll a; Chl b, chlorophyll b; Chl, total chlorophyll; Car, carotenoid.

With increasing drought severity, the contents of chlorophyll a, chlorophyll b, and total chlorophyll in the leaves of *D. chrysanthum* did not show significant differences among treatments (**Figure 3**). Likewise, the chlorophyll a/b ratio and carotenoid content remained statistically unchanged across treatments, and all parameters were maintained at comparable levels.

### 3.5 Anatomical characteristics

The measured anatomical traits showed different responses to drought stress (**Table 3**). RCT increased with increasing drought severity and was significantly higher under T2 than under CK and T1, whereas no significant difference was observed between CK and T1. RXVD decreased significantly under drought stress, with values under both T1 and T2 being significantly lower than those under CK, but not significantly different from each other. STAF decreased progressively with increasing drought intensity, following the order CK > T1 > T2, with significant differences among all treatments. In contrast, LT varied only slightly among treatments and showed no significant difference. These results indicate that drought stress significantly affected root cortex thickness, root xylem vessel diameter, and stem tissue structure, whereas leaf thickness remained relatively stable.

**Table 3:** Changes in anatomical structural parameters of roots, stems and leaves of *D. chrysanthum* under different drought stress treatments.

Treatment	RCT (µm)	RXVD (µm)	STAF (%)	LT (µm)
CK	249.77 ± 13.76^b^	29.97 ± 1.42^a^	98.17 ± 0.86^a^	237.28 ± 13.40^a^
T1	258.43 ± 32.28^b^	22.23 ± 0.78^b^	95.17 ± 1.06^b^	236.07 ± 6.23^a^
T2	323.83 ± 30.64^a^	23.07 ± 2.57^b^	92.17 ± 1.26^c^	232.03 ± 9.96^a^

Different lowercase letters within the same column indicate significant differences among treatments at *P* < 0.05, whereas the same lowercase letters indicate no significant difference. Data are presented as mean ± standard deviation (*n* = 4). RCT, root cortex thickness; RXVD, root xylem vessel diameter; STAF, stem tissue area fraction; LT, leaf thickness.

**Figure 4:**
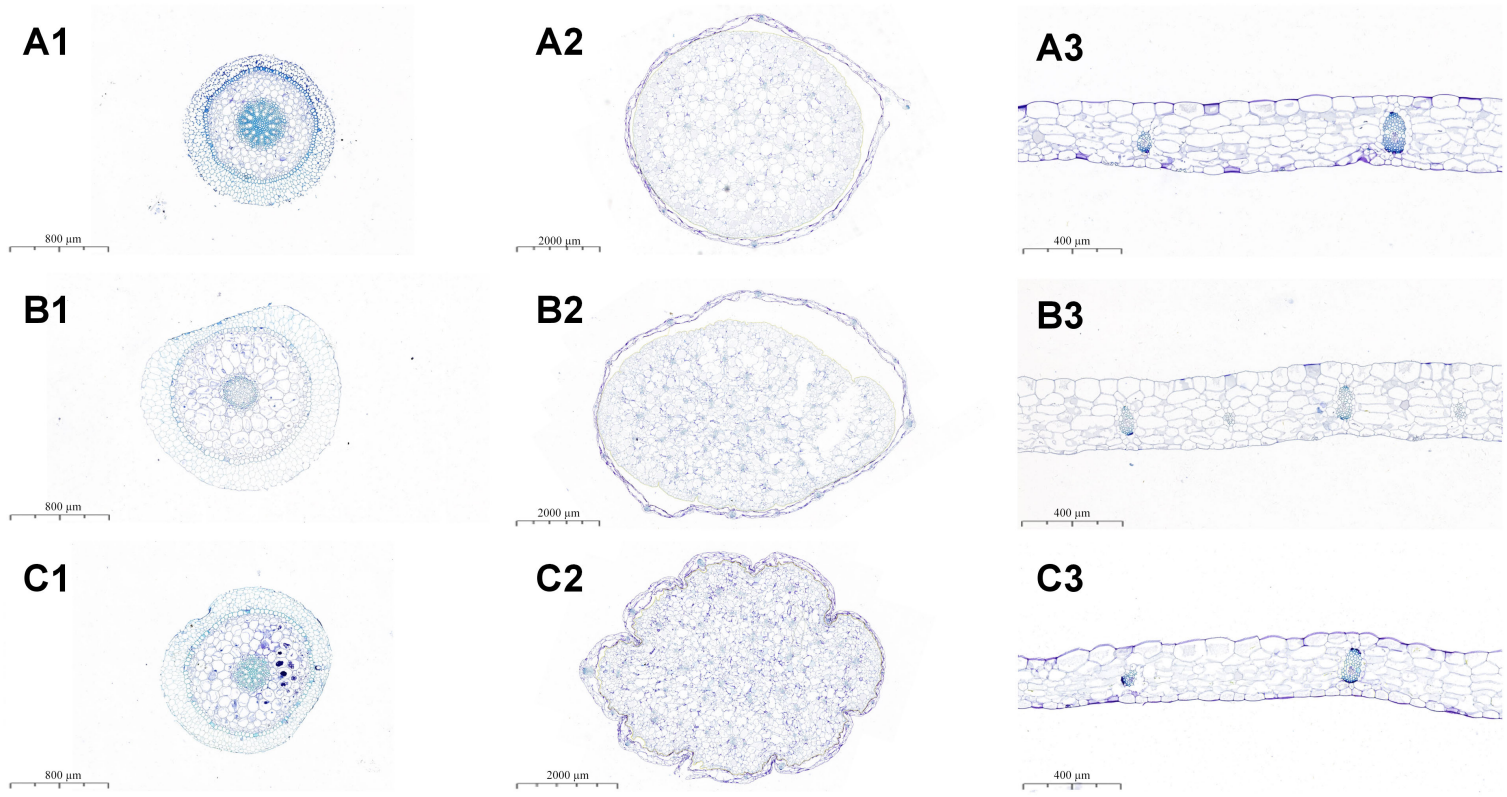
Anatomical cross-sections of roots, stems, and leaves of *D. chrysanthum* under different drought stress treatments. Different uppercase letters indicate different drought stress treatments (A: CK, B: T1, C: T2); different numbers represent different organs (1: root, 2: stem, 3: leaf). Scale bars: 800 µm (roots), 2000 µm (stems), 400 µm (leaves).

Microscopic observations were generally consistent with the quantitative results (**Figure 4**). In roots, no obvious tissue cavities were observed across treatments, but cortical parenchyma cells gradually shrank under drought stress, and root cross-sections became increasingly deformed from T1 to T2. In pseudobulbs, cortical parenchyma cells were turgid and closely arranged under CK, whereas they progressively lost turgor and became contracted as drought intensified. Although STAF decreased significantly from CK to T2, the values remained above 90% in all treatments because STAF represents a relative tissue area fraction rather than an absolute tissue area. Therefore, the moderate numerical decrease in STAF should be interpreted together with the microscopic observations, which showed evident shrinkage, deformation, and local enlargement of intercellular spaces in the cortical parenchyma under T2. In leaves, mesophyll tissues became slightly looser under drought stress, but no obvious change in tissue differentiation was observed, consistent with the non-significant change in LT. Overall, the statistical and microscopic results showed that roots and pseudobulbs exhibited more evident anatomical responses to drought stress than leaves.

### 3.6 Leaf epidermal characteristics

**Figure 5:**
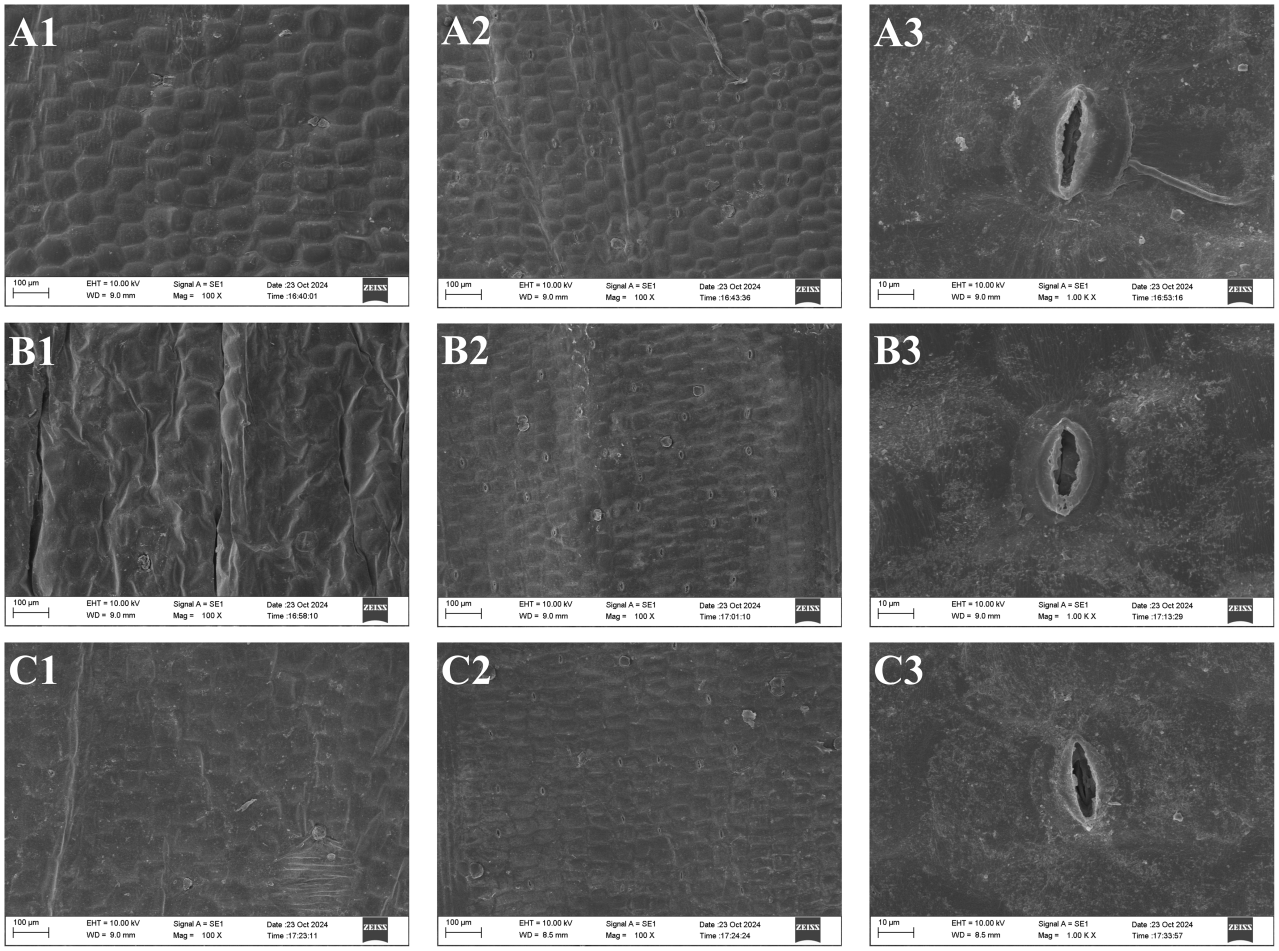
Scanning electron micrographs of leaf epidermal surfaces and stomata of *D. chrysanthum* under different drought stress treatments. Different uppercase letters indicate different drought stress treatments (A: CK, B: T1, C: T2); different numbers represent different structures (1: adaxial epidermis, 2: abaxial epidermis, 3: stomata). Scale bars: 100 µm (columns 1 and 2), 10 µm (column 3).

Leaf epidermal and stomatal traits exhibited distinct trends under drought stress (**Table 4**; **Figure 5**). Compared with CK, SD increased under both T1 and T2 treatments, although no significant difference was observed between the two drought treatments. SL and SW showed overall decreasing trends with increasing drought severity, with the smallest stomatal size observed under T2. Meanwhile, SA decreased progressively with drought stress and reached the lowest value under T2. Scanning electron microscopy revealed differences in stomatal morphology among treatments. Stomata in control plants were structurally intact, whereas those under drought stress appeared increasingly constricted, consistent with the quantitative changes observed.

**Table 4:** Changes in epidermal and stomatal characteristics of leaves in D. chrysanthum under different drought stress treatments.

Treatment	SD (ind.mm-2)	SL (µm)	SW (µm)	SA (µm2)
CK	51.33 ± 4.04^a^	24.83 ± 1.66^a^	4.21 ± 0.18^a^	88.03 ± 9.28^a^
T1	57.33 ± 4.34^a^	22.91 ± 2.24^ab^	3.95 ± 0.16^b^	74.61 ± 11.40^ab^
T2	57.67 ± 3.51^a^	21.11 ± 1.22^b^	3.55 ± 0.10^c^	58.65 ± 4.58^b^

Different lowercase letters within the same column indicate significant differences among treatments at *P* < 0.05, whereas the same lowercase letters indicate no significant difference. Data are presented as mean ± standard deviation (*n* = 4). SD, stomatal density; SL, stomatal length; SW, stomatal width; SA, stomatal area.

### 3.7 Osmotic adjustment substances

Drought stress induced distinct patterns of osmotic adjustment substances in stems and leaves of *D. chrysanthum* (**Table 5**). In stems, SP content increased significantly with drought severity and reached the highest level under T2. SS content was higher under drought treatments than under CK, with no significant difference between T1 and T2. ST content increased under T1 but decreased markedly under T2. In leaves, SP content was significantly higher under T2 than under CK and T1, whereas SS content showed relatively small variation among treatments. Leaf ST content increased significantly with increasing drought stress and reached its highest level under T2. These results demonstrate organ-specific differences in the direction and magnitude of osmotic adjustment responses under different drought intensities.

**Table 5:** Changes in osmotic adjustment substances in stems and leaves of *D. chrysanthum* under different drought stress treatments.

Organ	Treatment	SP (mg·g^-1^ FW)	SS (mg·g^-1^ FW)	ST (mg·g^-1^ FW)
Stem	CK	0.81±014^b^	10.76±089^b^	11..09±0.17^b^
	T1	1.11±0.10^b^	13.46±1.40^a^	13.64±0.95^a^
	T2	2.87±037^a^	14.83±1.56^a^	8.88±0.89^c^
Leaf	CK	3.48±0.26^b^	4.70±0.24^a^	3.60±0.23^c^
	T1	3.10±0.30^b^	4.08±0.20^b^	4.51±0.51^b^
	T2	4.31±0.03^a^	4.68±0.18^a^	5.74±0.49^a^

Different lowercase letters within the same column indicate significant differences among treatments at *P* < 0.05, whereas the same lowercase letters indicate no significant difference. Data are presented as mean ± standard deviation (*n* = 4). Units are expressed on a fresh-weight basis. SP, soluble protein; SS, soluble sugar; ST, starch.

### 3.8 Antioxidant enzyme activities

Antioxidant enzyme activities showed clear organ-specific responses to drought stress (**Table 6**). In stems, SOD and POD activities were strongly enhanced under T2, while CAT activity decreased under T1 and partially recovered under T2. In leaves, SOD activity increased markedly under T2, POD activity was higher under both drought treatments than under CK, and CAT activity decreased under drought stress. Overall, leaves showed higher antioxidant enzyme activities than stems, but pseudobulbs also displayed a pronounced enzymatic response under severe drought.

**Table 6:** Changes in antioxidant enzyme activities in stems and leaves of *D. chrysanthum* under different drought stress treatments.

Organ	Treatment	SOD (U·g^-1^ FW)	POD (U·g^-1^ FW)	CAT (nmol·min^-1^·g^-1^ FW)
Stem	CK	27.86 ± 1.60^b^	463.48 ± 19.57^b^	23.03 ± 5.15^ab^
	T1	28.11 ± 1.55^b^	644.61 ± 43.30^b^	17.40 ± 0.27^b^
	T2	125.46 ± 1.34^a^	2057.89 ± 121.02^a^	26.07 ± 0.49^a^
Leaf	CK	150.77 ± 9.43^b^	2369.80 ± 168.50^b^	46.55 ± 4.61a
	T1	123.87 ± 4.40^b^	3263.96 ± 307.34^a^	28.99 ± 4.79^b^
	T2	202.32 ± 24.29^a^	3048.96 ± 289.43^a^	35.25 ± 0.69^b^

Different lowercase letters within the same column indicate significant differences among treatments at *P* < 0.05, whereas the same lowercase letters indicate no significant difference. Data are presented as mean ± standard deviation (*n* = 4). Units are expressed on a fresh-weight basis. SOD, superoxide dismutase; POD, peroxidase; CAT, catalase.

### 3.9 Lipid peroxidation

**Figure 6:**
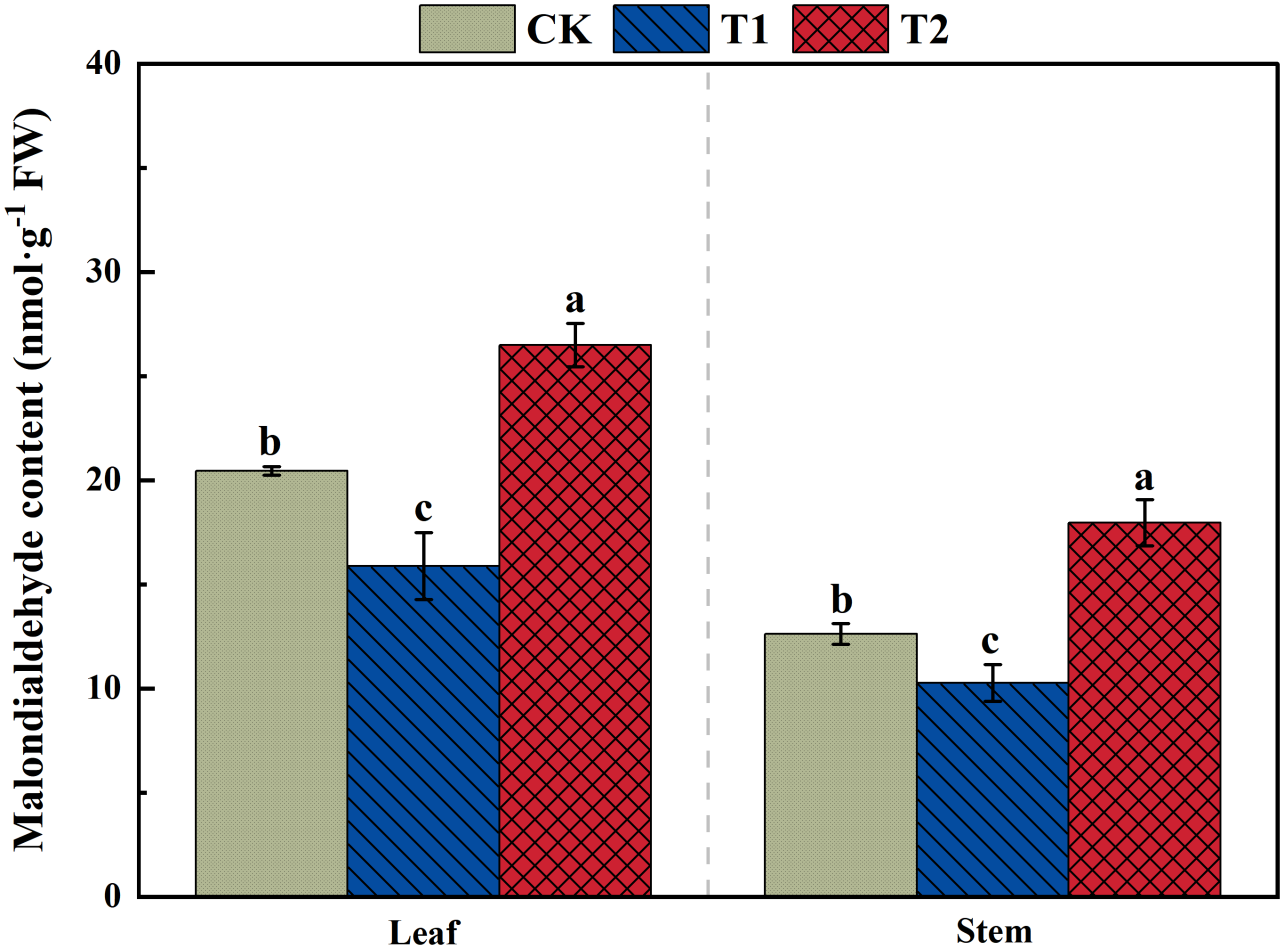
Changes in malondialdehyde (MDA) content in stems and leaves of *D. chrysanthum* under different drought stress treatments. Data are presented as mean ± standard deviation (*n* = 4). Different colored bars represent the control (CK), moderate drought stress (T1), and severe drought stress (T2), respectively. Different lowercase letters indicate significant differences among treatments at *P* < 0.05 within the same organ; the same letters indicate no significant difference.

MDA content in stems and leaves responded to drought stress in a consistent pattern, although with different magnitudes (**Figure 6**). Compared with CK, MDA content in both tissues decreased under T1 but increased markedly under T2. Comparisons between organs revealed that both stems and leaves exhibited a similar “decrease–increase” pattern across the drought gradient; however, leaf MDA content was consistently higher than that in stems and showed a more pronounced increase under T2. These results suggest that leaves may be more susceptible than stems to drought-induced lipid peroxidation, while stems appeared to maintain relatively stable membrane status under drought stress.

### 3.10 Correlation analysis

**Figure 7:**
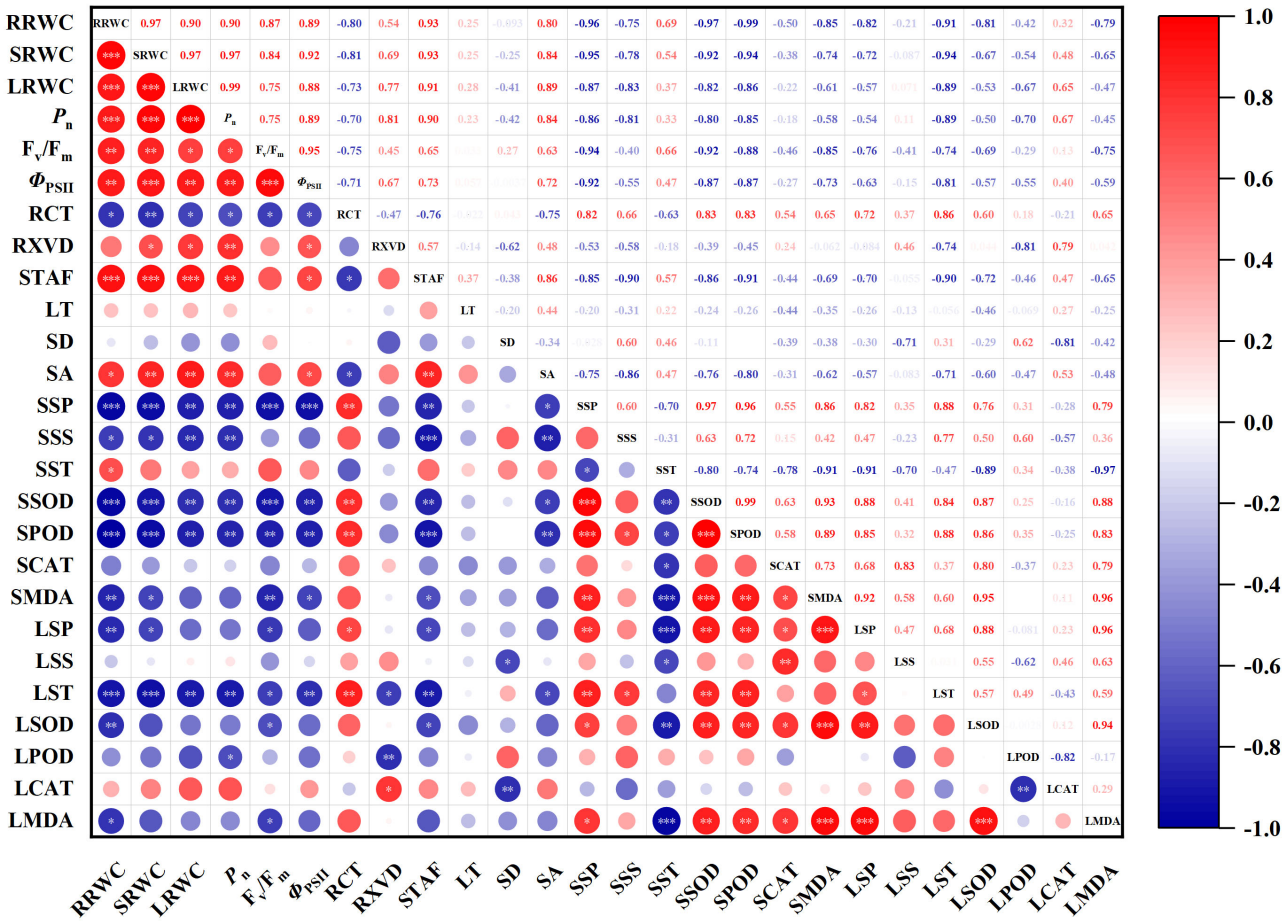
Pearson correlation heatmap among physiological, biochemical, and anatomical parameters of *D. chrysanthum* under different drought stress treatments. Circle size and color indicate the strength and direction of the correlation (red: positive; blue: negative). Numbers within circles represent Pearson correlation coefficients. Asterisks indicate statistical significance: *P < 0.05, **P < 0.01, ***P < 0.001. RRWC, root relative water content; SRWC, stem relative water content; LRWC, leaf relative water content; *P*_n_, net photosynthetic rate; *F*_v_/*F*_m_, maximum quantum efficiency of PSII; *Φ*PSII, effective quantum yield of PSII; RCT, root cortex thickness; RXVD, root xylem vessel diameter; STAF, stem tissue area fraction; LT, leaf thickness; SD, stomatal density; SA, stomatal area; SSP, stem soluble protein; SSS, stem soluble sugar; SST, stem starch; SSOD, stem superoxide dismutase activity; SPOD, stem peroxidase activity; SCAT, stem catalase activity; SMDA, stem malondialdehyde content; LSP, leaf soluble protein; LSS, leaf soluble sugar; LST, leaf starch; LSOD, leaf superoxide dismutase activity; LPOD, leaf peroxidase activity; LCAT, leaf catalase activity; LMDA, leaf malondialdehyde content.

Correlation analysis revealed clear relationships among physiological, biochemical, and anatomical parameters (**Figure 7**). Water status–related indicators (RRWC, SRWC, and LRWC) were significantly positively correlated with *P*_n_, *F*_v_/*F*_m_, and *Φ*PSII, and negatively correlated with MDA content and some antioxidant enzyme activities. Strong positive correlations were observed between photosynthetic and chlorophyll fluorescence parameters, particularly between *P*_n_ and both *F*_v_/*F*_m_ and *Φ*PSII. Antioxidant enzyme activities (SOD, POD, and CAT) showed overall positive correlations with MDA content and negative correlations with water status and photosynthetic parameters. Among anatomical traits, parameters such as root cortical thickness and leaf thickness were positively correlated with water status and photosynthetic parameters, whereas stomatal density and stomatal area exhibited certain correlations with gas exchange parameters. Collectively, these indicators formed an association pattern centered on water status, photosynthetic function, and oxidative stress, providing an integrative overview of trait coordination across the drought gradient.

## 4 Discussion

### 4.1 Water regulation strategies in epiphytic orchids

In drought-prone environments, the ability to acquire, store, and retain water is a fundamental determinant of plant survival (Chaves et al., 2003; Tardieu et al., 2018). As a typical epiphytic orchid, drought tolerance in *D. chrysanthum* does not rely on a single organ but emerges from functional differentiation and coordination among roots, pseudobulbs, and leaves. Previous studies on epiphytic orchids provide representative species-level evidence for this organ-level water-regulation strategy. For example, in *D. chrysotoxum* and *D. officinale*, water balance is maintained through two complementary strategies: thick leaf cuticles reduce water loss, whereas pseudobulbs provide internal water storage (Yang et al., 2016). In Pleione aurita, pseudobulbs have also been shown to store water and mobilize metabolic reserves during drought and subsequent recovery, indicating their role in both dehydration buffering and post-drought recovery (Zhang et al., 2018). These species-level examples support the view that water-conserving leaves and water-storing pseudobulbs are important components of drought adaptation in epiphytic orchids. Consistent with this framework, the present study revealed pronounced organ-specific differences in water responses under increasing drought stress. Although root RWC was relatively low in absolute terms, roots exhibited the smallest change from CK to T1, suggesting a role in early water-status stabilization. By contrast, pseudobulb RWC remained relatively high under moderate drought but declined markedly from T1 to T2, indicating delayed depletion of stored water during prolonged drought. Leaves showed the smallest overall decrease in RWC, suggesting that leaf water status was better maintained. After the drought treatment, plants were returned to normal watering and showed visible recovery of leaf posture and pseudobulb turgor within a short period. This observation suggests that the drought-induced dehydration symptoms were partly reversible at the morphological level, although the recovery of photosynthetic and biochemical functions requires further confirmation through formal rehydration experiments. Taken together, these results indicate a root–pseudobulb functional differentiation in *D. chrysanthum*, in which roots contribute mainly to early water-status stabilization, while pseudobulbs act as the main storage and buffering organs during prolonged drought. This organ-level division of labor reflects a structurally and functionally integrated adaptation to intermittently water-limited epiphytic habitats.

### 4.2 Drought responses of photosynthetic function

Drought stress generally exerts pronounced effects on plant photosynthesis by restricting gas exchange and impairing the structure and function of the photosynthetic apparatus (Xu et al., 2010; Qiao et al., 2024). In the present study, both the *P*_n_ and *T*_r_ of *D. chrysanthum* decreased under drought treatments; however, under moderate drought (T1) the plants were still able to maintain a certain level of photosynthetic activity, indicating a degree of regulatory flexibility of the photosynthetic system. This response is closely related to the ecological background of epiphytic orchids, which have long adapted to intermittent water supply and whose photosynthetic strategy emphasizes stability rather than maximum efficiency (Rodrigues et al., 2013; Zhang et al., 2018). Under severe drought (T2), *P*_n_ declined sharply and became slightly negative, whereas the intercellular CO_2_ concentration (*C*_i_) increased, showing an opposite trend. This divergence indicates that the limitation to photosynthesis was no longer mainly caused by stomatal closure and restricted CO_2_ diffusion, but was instead dominated by non-stomatal factors associated with reduced mesophyll carbon assimilation capacity. Under intense drought, decreases in Rubisco activity, inhibition of key enzymes in the Calvin cycle, and insufficient energy supply from photochemical reactions can all reduce CO_2_ fixation efficiency, resulting in the accumulation of CO_2_ in the intercellular spaces and the characteristic pattern of elevated *C*_i_ concomitant with strongly suppressed *P*_n_. These results suggest that, at the T2 drought level, the photosynthetic limitation in *D. chrysanthum* shifts from stomatal regulation to impairment of biochemical processes and photosystem function, reflecting substantial inhibition of the photosynthetic machinery by severe water deficit. Although photosynthetic pathway plasticity along the C_3_–CAM continuum has been reported in some epiphytic orchids, the gas-exchange responses observed in the present study should be interpreted cautiously. A species-specific study reported that *D. chrysanthum* exhibits C_3_-type leaf anatomical and physiological characteristics, including no nocturnal CO_2_ uptake, low PEPCase activity, and relatively high RuBPCase and glycolate oxidase activities, indicating that this species belongs to the C_3_ photosynthetic type (Zhu et al., 2013). Therefore, because *δ*^13^C values, nocturnal stomatal conductance, and diel acid fluctuation were not measured in the present study, we do not infer CAM induction or a C_3_–CAM transition under drought. Instead, the decline of daytime *P*_n_ to slightly negative values under T2, together with the increase in *C*_i_ and the sharp reduction in *G*_s_, is more likely to reflect severe non-stomatal inhibition of carbon assimilation, reduced carboxylation capacity, and a net carbon balance in which respiratory and maintenance carbon costs exceeded daytime carbon gain under prolonged water deficit. Ecologically, this response suggests that *D. chrysanthum* had shifted from a growth-oriented carbon-gain strategy to a survival-maintenance state under severe drought. Such photosynthetic down-regulation may help reduce water loss and metabolic demand in the short term, but prolonged negative carbon balance would constrain growth, recovery, and population persistence under increasingly frequent drought events.

Unlike the marked declines observed in gas-exchange parameters and *Φ*_PSII_, photosynthetic pigment contents in *D. chrysanthum* remained relatively stable on a fresh-weight basis. Chl a, Chl b, total chlorophyll, carotenoid contents, and the Chl a/b ratio showed no significant differences among CK, T1, and T2, indicating that drought-induced photosynthetic inhibition was not mainly caused by pigment loss or major remodeling of the light-harvesting pigment composition. Instead, the decline in carbon assimilation and PSII performance was more closely associated with stomatal restriction under moderate drought and functional photochemical inhibition under severe drought. The maintenance of pigment contents may represent a conservative protective strategy that preserves light-harvesting capacity while photosynthetic activity is down-regulated during water deficit. Similar maintenance of pigment pools under moderate drought has been reported in epiphytic orchids and may reflect the combined effects of structural water buffering and antioxidant protection (Rodrigues et al., 2013; Yang et al., 2016). Therefore, the decline in *F*_v_/*F*_m_ and *Φ*_PSII_ under severe drought should be interpreted primarily as functional photochemical down-regulation rather than as a consequence of substantial pigment degradation.

### 4.3 Structural regulation through anatomy and stomatal traits

Plant microstructural traits constitute an essential component of drought adaptation, as stomatal structure and distribution directly determine leaf water loss and gas exchange capacity, while the development of water-storage tissues is critical for buffering and maintaining plant function under fluctuating water availability (Hetherington and Woodward, 2003; Woning et al., 2025). The present study shows that *D. chrysanthum* exhibits pronounced anatomical adjustments under drought stress, particularly in stomatal characteristics and water-storage structures. Compared with many soil-rooted plants, epiphytic orchids rely more strongly on organ-level water storage and stomatal adjustment because their water supply is intermittent and weakly buffered by substrate moisture. Such moderate stomatal regulation can effectively decrease transpiration while maintaining sufficient CO_2_ uptake to sustain basic photosynthetic activity (Drake et al., 2013; Lawson and Blatt, 2014), thereby avoiding the negative effects of excessive stomatal closure on carbon assimilation (Buckley, 2019). In addition, the increased thickness of the root cortex, the enhanced compactness of stem tissues, and the relatively loose arrangement of leaf mesophyll collectively contribute to drought adaptation (Sack and Holbrook, 2006; Scoffoni et al., 2018). Thickened root cortex favors short-term water storage, whereas the compact stem structure helps reduce transpirational water loss (North and Nobel, 1997). The relatively uniform and weakly differentiated mesophyll in leaves may also minimize internal water potential gradients and prevent localized dehydration (Zhang et al., 2015). Compared with other epiphytic plants, the anatomical configuration of *D. chrysanthum* thus confers distinct advantages in stomatal regulation, water storage, and restriction of water loss, further enhancing its capacity to survive under drought conditions (Batke et al., 2018).

### 4.4 Coordinated regulation of osmotic adjustment and antioxidant defense

Under drought stress, plants face not only water deficit but also osmotic imbalance and excessive ROS accumulation (Krasensky and Jonak, 2012). The present study demonstrates that D. chrysanthum counteracts these stresses through coordinated osmotic adjustment and antioxidant defense. The accumulation of soluble sugars, soluble proteins, and starch in stems and leaves likely contributed to osmotic regulation and mitigation of dehydration-induced cellular stress (Thalmann and Santelia, 2017; Ozturk et al., 2021), with the pseudobulb acting as an important organ for water storage and metabolic buffering. Antioxidant enzyme activities also showed organ-specific responses to drought stress. In stems, SOD activity increased significantly only under severe drought, while POD activity increased progressively and CAT activity recovered under T2 after a decline under T1. In leaves, SOD and POD activities remained relatively high and were further enhanced under drought stress, whereas CAT activity decreased under both drought treatments. These results suggest that D. chrysanthum activated different antioxidant strategies in stems and leaves to cope with drought-induced oxidative pressure.

MDA content showed a nonlinear response in both leaves and stems. Under moderate drought, MDA content decreased significantly in both organs, suggesting that moderate water deficit did not enhance membrane lipid peroxidation and may have induced protective metabolic adjustment. However, under severe drought, MDA content increased markedly in both leaves and stems, indicating that prolonged water deficit may have exceeded the protective capacity of osmotic and antioxidant regulation, resulting in enhanced membrane lipid peroxidation (Apel and Hirt, 2004). Although stem MDA also increased under severe drought, its absolute level remained lower than that in leaves across all treatments. This pattern suggests that leaves may be more susceptible to drought-induced lipid peroxidation, whereas pseudobulbs may retain a relatively stronger buffering capacity against oxidative damage. However, because ROS levels, membrane integrity, and cellular oxidative damage were not directly measured in this study, this interpretation should be regarded as a plausible explanation rather than direct mechanistic evidence. Further studies combining ROS localization, membrane stability assays, and histochemical detection are needed to clarify the role of pseudobulbs in oxidative stress mitigation under drought.

### 4.5 Integration of drought responses based on correlation analysis

Correlation analysis provided an integrative overview of the associations among water status, photosynthetic, biochemical, and structural traits in *D. chrysanthum* across the drought gradient. Strong positive correlations between RWC indicators and photosynthetic parameters indicate that the maintenance of organ water status was closely associated with photosynthetic stability under drought stress. Meanwhile, the correlations among osmotic adjustment substances, antioxidant enzyme activities, and MDA suggest that osmotic regulation and ROS-scavenging responses were associated with changes in membrane lipid peroxidation (Foyer and Noctor, 2005; Nakabayashi and Saito, 2015). These relationships support the view that drought adaptation in *D. chrysanthum* involves multi-level coordination among water regulation, photosynthetic down-regulation, structural adjustment, and biochemical defense. However, because the correlation analysis was performed using pooled data from all treatments, it should be interpreted as reflecting overall associations across the drought gradient rather than treatment-specific regulatory relationships. Thus, the correlation results do not indicate how these relationships changed within CK, T1, or T2 separately, but they provide a useful framework for identifying the major trait modules associated with drought response in this species. This integrated strategy highlights the adaptive significance of root–pseudobulb–leaf functional integration in epiphytic orchids, whose water availability is intermittent and less buffered by soil water reserves than that of many ground-growing plants (Cernusak, 2020). Given the limited sample size and the use of pooled data across treatments, the Pearson correlation results should be interpreted as exploratory, gradient-level associations rather than causal or treatment-specific relationships.

### 4.6 Ecological specificity and conservation implications

Compared with other *Dendrobium* species examined under drought conditions, *D. chrysanthum* shows broadly similar organ-level regulatory patterns—including pseudobulb water buffering, osmotic adjustment, and antioxidant activation—to those reported in *D. officinale* and *D. huoshanense* (Yang et al., 2016), suggesting that these strategies represent conserved drought-response features within the genus. Similarly, *D. nobile* and related congeners have been reported to sustain basic photosynthetic function and membrane integrity under moderate drought through coordinated pseudobulb water buffering and enzymatic antioxidant upregulation (Stancato et al., 2001; Rodrigues et al., 2013), further supporting the view that pseudobulb-mediated homeostasis is a shared adaptive feature of epiphytic *Dendrobium*.

Nevertheless, the drought-response strategy of *D. chrysanthum* appears to be calibrated for the relatively humid montane monsoon forests it naturally inhabits, where inter-rainfall intervals are typically shorter than those experienced by species distributed in more xeric epiphytic habitats. Although direct field-monitoring data on the frequency of water stress experienced by wild *D. chrysanthum* populations are currently lacking, its epiphytic growth on tree trunks and rock surfaces suggests that plants are likely exposed to repeated short-term dry episodes during rainless intervals, especially after the rainy season or on more exposed microsites with limited external water retention. Consequently, while *D. chrysanthum* possesses effective short-term buffering mechanisms, its physiological homeostasis deteriorated markedly under 28 days of continuous water withholding, suggesting a relatively narrow drought-tolerance window compared with congeners adapted to more episodic water regimes. This ecological sensitivity likely reflects species-specific variation in pseudobulb water-storage capacity and the efficiency of osmotic adjustment, rather than a fundamental absence of drought-adaptive mechanisms.

From a conservation perspective, the ecological sensitivity of *D. chrysanthum* to prolonged drought underscores its vulnerability to the increasing frequency and duration of extreme drought events associated with climate change (Dai, 2013; Cook et al., 2020). Given that wild populations of this species are already under pressure from habitat loss, deforestation, and overcollection (Swarts and Dixon, 2009), projected shifts in precipitation regimes may further constrain its distribution and reproductive success in native habitats. These findings therefore highlight the practical importance of maintaining adequate and consistent water supply in *ex situ* cultivation and reintroduction programs, particularly by avoiding prolonged continuous water deficit beyond the short-term dry intervals that this species is likely to encounter in its natural epiphytic habitats. Irrigation scheduling should be designed to avoid continuous water deficit exceeding two weeks during the growing season. Although plants showed visible recovery of leaf posture and pseudobulb turgor after normal watering was resumed, this observation only indicates partial morphological recovery. Whether photosynthetic function and biochemical homeostasis can fully recover after drought requires further confirmation through formal drought–rehydration experiments.

## 5 Conclusions

This study provides a systematic, multi-level characterization of the drought-adaptation strategy of the endangered epiphytic orchid *D. chrysanthum*. Under progressive water deficit, the larger decline in stem RWC than in leaf RWC suggests that pseudobulbs may contribute to buffering leaf dehydration, although direct evidence for stem-to-leaf water redistribution was not obtained. Photosynthetic limitation shifted from stomatal to non-stomatal as drought intensified: under moderate drought, gas exchange was suppressed while PSII structural integrity and photosynthetic pigments were largely preserved; under severe drought, ΦPSII and Fv/Fm declined markedly, indicating partial photoinhibition. Microstructural adjustments, including increased root cortex thickness, reduced xylem vessel diameter, and decreased stem tissue area fraction, collectively reflected structural regulation associated with water conservation and hydraulic adjustment. At the biochemical level, organ-specific changes in osmolytes and antioxidant enzymes (SOD, POD, and CAT) contributed to drought-induced metabolic regulation. MDA content increased under severe drought in both leaves and stems, indicating enhanced lipid peroxidation, while its consistently lower level in stems than in leaves suggests that pseudobulbs may retain a relatively stronger buffering capacity against oxidative damage. Collectively, *D. chrysanthum* appears to employ a conservative, pseudobulb-associated drought-response strategy that integrates organ-specific structural, photosynthetic, and biochemical adjustments under drought stress. Given the marked inhibition observed under severe drought, maintaining adequate water supply is critical for the cultivation and *ex situ* conservation of this species under increasingly variable precipitation regimes.

## Data Availability

The original contributions presented in the study are included in the article/supplementary material. Further inquiries can be directed to the corresponding authors.
